# The clinical effects of pulsed electromagnetic field therapy for the management of chronic ankle instability: a study protocol for a double-blind randomized controlled trial

**DOI:** 10.1186/s13063-024-08639-z

**Published:** 2024-12-03

**Authors:** Cheryl Shu Ming Chia, Sai-Chuen Fu, Xin He, Yang Yang Cheng, Alfredo Franco-Obregón, Yinghui Hua, Patrick Shu-Hang Yung, Samuel Ka-Kin Ling

**Affiliations:** 1 Department of Orthopaedics and Traumatology, Faculty of Medicine, The Chinese University of Hong Kong (CUHK), Sha Tin, Hong Kong, China; 2 Department of Health Technology and Informatics, The Hong Kong Polytechnic University, Hung Hom, Hong Kong, China; 3 Department of Surgery, Yong Loo Lin School of Medicine, National University of Singapore, Singapore, 119228 Singapore; 4 Institute of Health Technology and Innovation (iHealthtech), National University of Singapore, Singapore, 117599 Singapore; 5 Biolonic Currents Electromagnetic Pulsing Systems Laboratory (BICEPS), National University of Singapore, Singapore, 117599 Singapore; 6 Department of Physiology, Yong Loo Lin School of Medicine, National University of Singapore, Singapore, 117593 Singapore; 7 Healthy Longevity Translational Research Programme, Yong Loo Lin School of Medicine, National University of Singapore, Singapore, 119228 Singapore; 8 Nanomedicine Translational Research Programme, Centre for NanoMedicine, Yong Loo Lin, School of Medicine, National University of Singapore, Singapore, Singapore; 9 NUS Centre for Cancer Research, Yong Loo Lin School of Medicine, National University of Singapore, Singapore, 117599 Singapore; 10 Department of Sports Medicine, Huashan Hospital, Fudan University, Shanghai, China

**Keywords:** Chronic ankle instability, Pulse electromagnetic field, Ankle sprain, Anterior talofibular ligament, Randomized controlled trial

## Abstract

**Background:**

Chronic ankle instability is associated with long-term neuromuscular deficits involving poor postural control and peroneal muscular impairment. Symptoms of chronic ankle instability hinder engagement in physical activity and undermine the patient’s quality of life. Despite the existence of various treatment modalities, none has conclusively provided evidence of clinical effectiveness in counteracting neuromuscular deficits, such as arthrogenic muscle inhibition of the peroneal longus (PL). Pulse electromagnetic field therapy employed as an adjunct biophysical therapy can potentially improve stability by mitigating peroneal muscle weakness and by activating the peroneal muscle. We postulate that by combining standard care (muscle strengthening, balance training, and range of motion exercise) with pulse electromagnetic field therapy, postural control stability and peroneal muscle weakness will significantly improve.

**Methods:**

This is a prospective, randomized, double-blind, placebo-controlled trial. A total of 48 adults with chronic ankle instability will be recruited and randomly allocated into either the intervention or control groups. The intervention group (*n* = 24) will receive active pulse electromagnetic field therapy and standard exercise training, while the control group (*n* = 24) will receive sham pulse electromagnetic field therapy and standard exercise training for 8 weeks. Primary and secondary outcomes will be evaluated at baseline, week 4, 8 as well as at 3-, 6-, and 12-month follow-up visits.

**Discussion:**

Chronic ankle instability is a common debilitating condition without a curative conservative treatment. Investigating different treatment modalities will be essential for improving rehabilitation outcomes in this clinical population. This study will investigate the effectiveness of pulsed electromagnetic field therapy on the functional and clinical outcomes in the chronic ankle instability population. This trial may demonstrate this non-invasive biophysical therapy to be an effective measure to help patients with CAI.

**Trial registration:**

ClinicalTrials.gov NCT05500885. Registered on August 13, 2022.

## Administrative information

Note: the numbers in curly brackets in this protocol refer to SPIRIT checklist item numbers. The order of the items has been modified to group similar items (see http://www.equator-network.org/reporting-guidelines/spirit-2013-statement-defining-standard-protocol-items-for-clinical-trials/).

**Table:** 

Title {1}	Study protocol for a double-blinded randomized controlled trial on the clinical effectiveness of pulsed electromagnetic field therapy for the management of chronic ankle instability
Trial registration {2a and 2b}	ClinicalTrials.gov NCT05500885 registered on 13 August 2022
Protocol version {3}	Version 1
Funding {4}	No funding
Author details {5a}	Shu Ming Cheryl Chia, MSc^1^, Sai-Chuen Fu, PhD^1^, Xin He, PhD^1^, Yang Yang, Mphil^2^, Alfredo Franco-Obregón, PHD^3^, Yinghui Hua, MD^4^, Patrick Shu-Hang Yung, MBChB^1^, Samuel Ka-Kin Ling, MBChB^1^ ^1^Department of Orthopaedics and Traumatology, Faculty of Medicine, The Chinese University of Hong Kong (CUHK), Hong Kong SAR, China ^2^Department of Health Technology and Informatics, The Hong Kong Polytechnic University, Hong Kong SAR, China ^3^Department of Surgery, Yong Loo Lin School of Medicine, National University of Singapore, 119,228, Singapore; Institute of Health Technology and Innovation (iHealthtech), National University of Singapore, 117,599, Singapore; Biolonic Currents Electromagnetic Pulsing Systems Laboratory (BICEPS), National University of Singapore, 117,599, Singapore; Department of Physiology, Yong Loo Lin School of Medicine, National University of Singapore, 117,593, Singapore; Healthy Longevity Translational Research Programme, Yong Loo Lin School of Medicine, National University of Singapore, 119,228, Singapore; Nanomedicine Translational Research Programme, Centre for NanoMedicine, Yong Loo Lin School of Medicine, National University of Singapore, Singapore; NUS Centre for Cancer Research, Yong Loo Lin School of Medicine, National University of Singapore, 117,599, Singapore ^4^Department of Sports Medicine, Huashan Hospital, Fudan University, Shanghai, China
Name and contact information for the trial sponsor {5b}	The Chinese University of Hong Kong Department of Orthopaedics and Traumatology Dr. Samuel Ka-Kin Ling Room 74,034, 5/F, Lui Che Woo Clinical Science Building, Prince of Wales Hospital, Shatin, Hong Kong SAR, Chinasamuel.ling@link.cuhk.edu.hk
Role of sponsor {5c}	The study sponsor takes on the legal responsibility for the initiation and management of the research study, including study design, data collection and analysis, the safety of participants, and publication.

## Introduction

### Background and rationale {6a}

Lateral ankle sprain (LAS) is one of the most common musculoskeletal injuries, with reports of 7 ankle sprains per 1000 exposures [1]. LAS is not merely an isolated ligamentous injury to the anterior talofibular ligament or calcaneofibular ligament but involves damage to the adjacent structures such as the peroneal longus (PL), which are one of the main ankle muscles essential for maintaining mediolateral stability [2]. Approximately 60% of individuals who sustained an acute LAS do not seek appropriate medical advice [3], potentially exacerbating the development of chronic ankle instability (CAI). CAI currently affects up to 40% of all individuals who have sustained one LAS, and the overall prevalence of CAI is reported to be 46%, ranging between 9 and 76% [4, 5].

CAI is a debilitating condition characterized by persistent symptoms (> 1 year after the index injury) of pain, swelling, diminished ankle range of motion (DROM), recurrent sprains, impaired function, and muscle weakness [4]. CAI hinders individuals from engaging in physical activity and reduces their overall quality of life [6]. It may also predispose to the development of post-traumatic ankle osteoarthritis leading to permanent dysfunction [7, 8]. Because of its high incidence, CAI is a social-economic and health burden estimated to cost ~ USD $11 billion annually, up from 38.2% since 2000 [9].

Muscle strengthening, balance training, and range of motion exercise remain the core tenets of standard care (SC) for CAI. The available evidence that rehabilitative exercise is capable of relieving pain, improving function, and decreasing recurrent ankle sprain is overall superior to a wait-and-see approach; however, the overall effectiveness of SC was low to moderate and inconsistent [10, 11]. A recent review has highlighted that the lone muscle strength training regime did not exert any clinical improvement in dynamic balance or self-reported function [12]. Moreover, in a prospective study, supervised balance training did not demonstrate any long-term improvements in muscle strength and dynamic balance among patients with CAI [13]. It has been suggested that 70% of individuals with CAI do not respond well to rehabilitation [14, 15]. Hence, a novel treatment is urgently warranted in addition to the SC. Overall, although universally adopted, the efficacy of SC remains conflicting and there is an ongoing search for novel treatment strategies with which to target the continuum of neuromuscular impairment and disability in patients with CAI.

Pulsed electrical magnetic field (PEMF) therapy is a biophysical therapeutic modality that has been shown to improve muscle regeneration, reduce pain, and attenuate inflammation in various musculoskeletal disorders and aging [16–19]. Preliminary evidence has demonstrated that PEMF therapy can enhance muscle performance, attributed to the activation of type I and IIa muscular fibers, similar to exercise [20]. The mechanism of PEMF therapy involves the activation of the transient receptor potential canonical-1 mediated calcium channel entry and down-stream regulatory factor, peroxisome proliferator-activated receptor-gamma coactivator (PGC-1α) which modulates mitochondrial activities and muscle contraction, respectively [21–23]. Mainly, PGC-1α activates muscle contraction and skeletal muscle fiber transformation through Ca^2+^/calmodulin-dependent protein kinase IV [24].

Furthermore, unlike electrical stimulation therapies where electrical current travels linearly and superficially from one electrode to the other along the surface of the muscle, the magnetic fields penetrate throughout the muscle with little obstruction in all three dimensions within the volume of the coil system wherein the muscle is contained and without the sensation of electric shock caused by the activation of nociceptors [25]. Indeed, PEMF therapy is commonly associated with analgesic effects and does not induce pain [19, 26–29].

To the best of our knowledge, this is the first clinical trial that investigates the effectiveness of PEMF therapy in the short and long term regarding the clinical and functional outcomes of CAI. In this trial, a rigorous double-blind, randomized controlled trial will be conducted to observe the effects of PEMF therapy in addition to the SC on clinical and functional aspects of CAI. The findings of this study will fill the research gap in the efficacy of PEMF therapy for the treatment of CAI.

### Objectives {7}

The primary objective is to investigate the clinical effects of PEMF therapy and SC on the clinical and functional outcomes in the CAI population. We hypothesize that the combination of PEMF therapy and SC will effectively improve the clinical and functional outcomes of patients with CAI.

### Trial design {8}

**Fig. 1:**
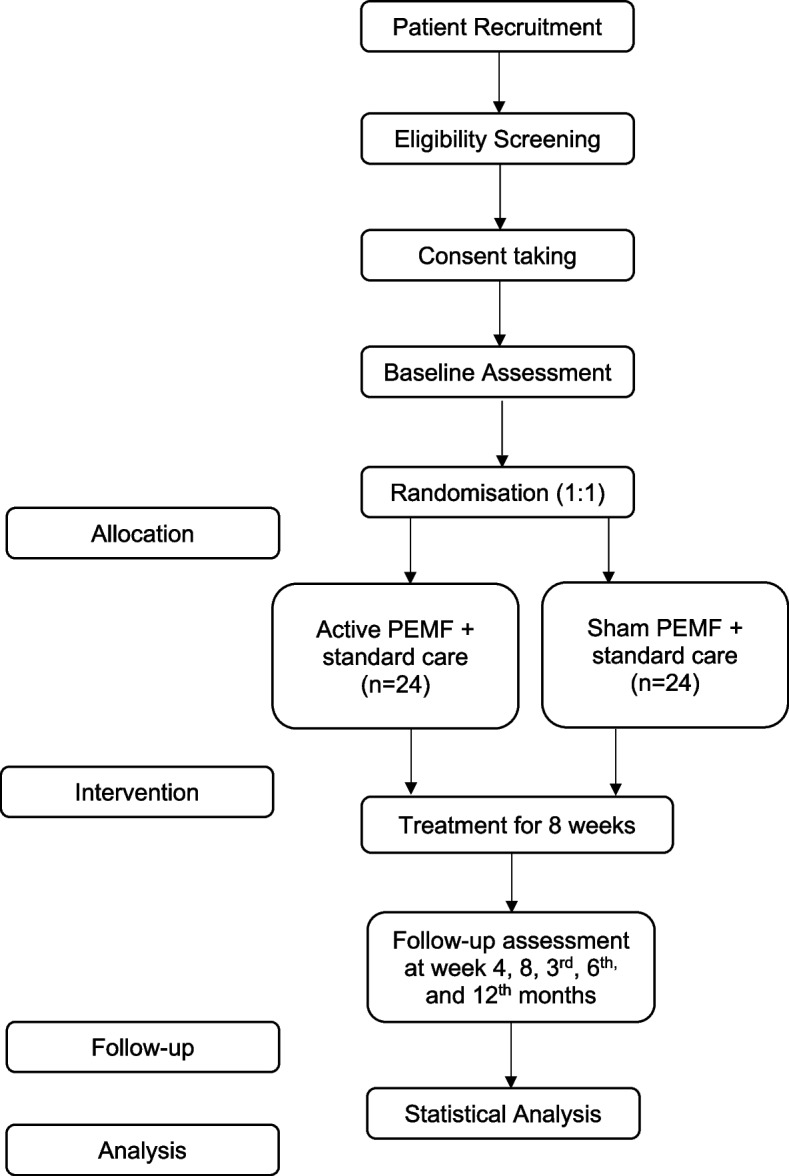
Overview of the study

This is a prospective randomized, double-blind, placebo-controlled trial with two parallel groups and a 1:1 allocation ratio to investigate the treatment effects of PEMF for participants with CAI. All participants will be randomized into an intervention group (*n* = 24, active PEMF and SC) and the control group (*n* = 24, sham PEMF and SC). Researchers will perform all patient-reported outcome measures (PROM), functional, and ultrasonographic outcomes during baseline assessment, 4 weeks, 8 weeks, and follow-up visits on the 3rd month, 6th month, and 1 year after the commencement of the trial. An overview of the study is displayed in Fig. 1.

## Methods: participants, interventions, and outcomes

### Study setting {9}

This study will be conducted at the Sports Performance and Biomechanics Laboratory at the Prince of Wales Hospital teaching hospital, the Chinese University of Hong Kong (CUHK).

### Eligibility criteria {10}

The eligibility criteria will follow the recommendations of the International Ankle Consortium [30].

Inclusion criteria:

1. Age between 18 and 60 years old.
2. A history of at least 1 significant ankle sprain.
  The initial sprain must have occurred 12 months before the study enrolment, be associated with pain and swelling, and have interrupted at least 1 day of the desired physical activity.
3. A history of the previously injured ankle joint having “given way,” recurrent sprain, and “feelings of instability.”
4. Cumberland Ankle Instability Tool (CAIT) score ≤ 24.
5. In bilateral cases, the ankle with a lower CAIT score will be considered for treatment.

Exclusion criteria:

1. History of major limb injury or surgery.
2. Other concomitant lower extremity pathology (e.g., osteoarthritis).
3. Other muscular, joint, or neurological impairments (e.g., dementia, cerebrovascular accidents) that affect lower limb functions and hinder the participants from completing the assessments.
4. Pregnant or planning for pregnancy.
5. Metal implantation in their body (e.g., pacemakers).

### Who will take informed consent? {26a}

After the eligible participants have been screened based on the inclusion and exclusion criteria, the orthopedic surgeon or the research team will explain the procedure, the benefits, potential risks, and the confidentially of the information and then obtain their signed consent.

### Additional consent provisions for collection and use of participant data and biological specimens {26b}

The written consent form will permit the researchers to collect and analyze the participant data. All data will be kept for a maximum 5 years after publication and destroyed after that time. The collected data will be anonymously published to the public through conferences and scientific papers. This study does not require any use of biological specimens.

## Interventions

### Explanation for the choice of comparators {6b}

Participants will be randomized into either the intervention group involving the active PEMF therapy and SC regime or the control group, receiving a sham exposure from the same PEMF device and SC regime.

### Intervention description {11a}

Participants randomized into the experimental group will be exposed to PEMF therapy employing a BIXEPS device (Quantum TX Pte. Ltd., Singapore) as previously described by Venugobal et al. [19]. Exposure will be given twice a week for 8 weeks for a total of 16 sessions on their affected foot in adjunct with SC. Each treatment session will last for 10 min. The active PEMF therapy does not produce any sensation, facilitating the blinding process. Meanwhile, the control group will receive the SC and the sham treatment from the same PEMF device. A team researcher will operate the PEMF device and monitor any clinical changes during the randomized clinical trial. Each participant will be tagged with a unique radiofrequency identification (RFID) card supplied by Quantum TX Pte. Ltd. using block randomization to assign the treatment group. To mitigate any selection bias, the block size will be concealed from the investigators, and all will be blinded from the subjective assignment.

A team researcher will be responsible for turning on the PEMF machine for all the participants using the RFID cards. A biostatistician not involved in the participant recruitment will oversee the randomization procedure. Both the research team and the biostatistician will remain blinded during the trial. Participants will use the RFID card to complete the treatment without knowledge of group allocation. Side effects will be reported if there are any.

**Fig. 2:**
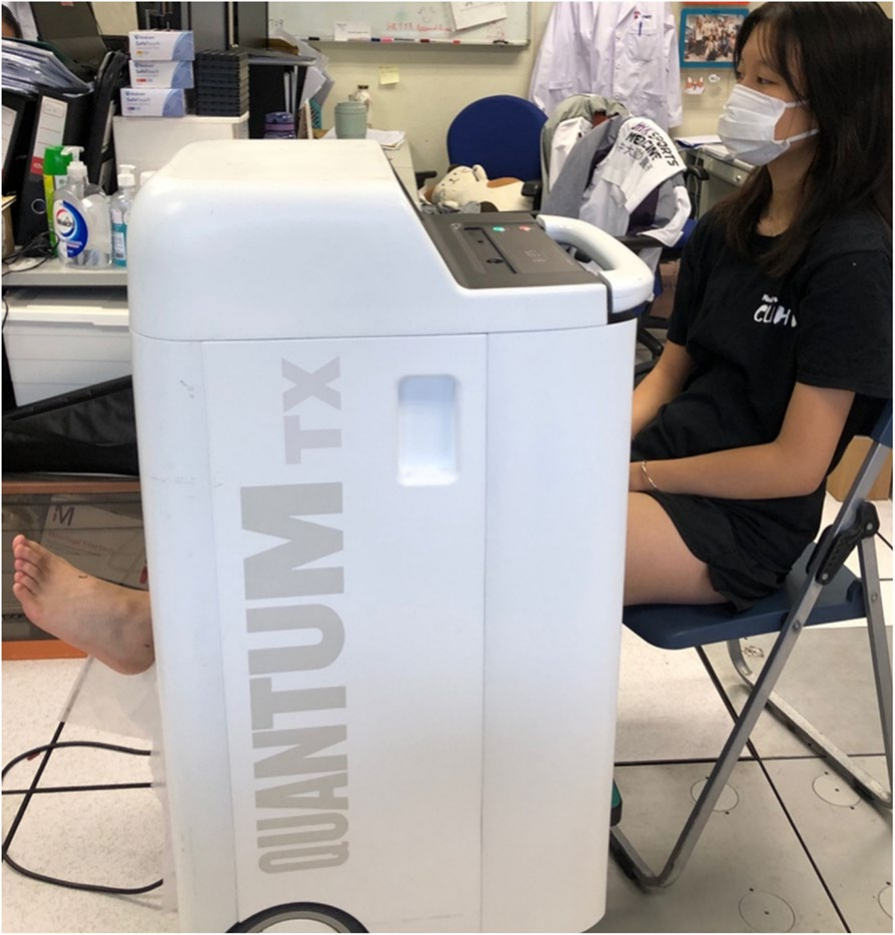
Demonstration on participant being treated with PEMF therapy

The treatment procedure requires that the participant be seated at 90 degrees on a chair. The solenoids will be adjusted to cover the lower limb. The BIXEPS device is set to low frequency and amplitude fields (1 mT and 50 Hz) to be applied to the affected limb for 10 min. Sham PEMF therapy and SC will be used for the control group. Since the PEMF therapy does not elicit any sensation, both groups are blinded, and the same SC regime will be administered to both groups. Figure 2 depicts how the participants will be situated relative to the BIXEPS device.

The administered SC exercise regime to the participants will be adapted from Alahmari et al. [31] with a detailed regime made into a booklet for the participants. Participants must conduct a warm-up (stretching around the ankle joint) for 5 min before the exercise regime. Participants will be required to engage between the first and 3rd week; participants will be required to participate in balance training while standing on an unstable surface between the first and third week. The treatment regime consists of 5 times/week for 15 to 20 min a day. During the wobble-board training, participants must move from front to back, left to right side for 15 s, 10 repetitions, with 10 s of rest between each repetition. Participants are later required to move in a circle for 60 s, 5 repetitions, with a rest of 20 s between each repetition.

**Fig. 3:**
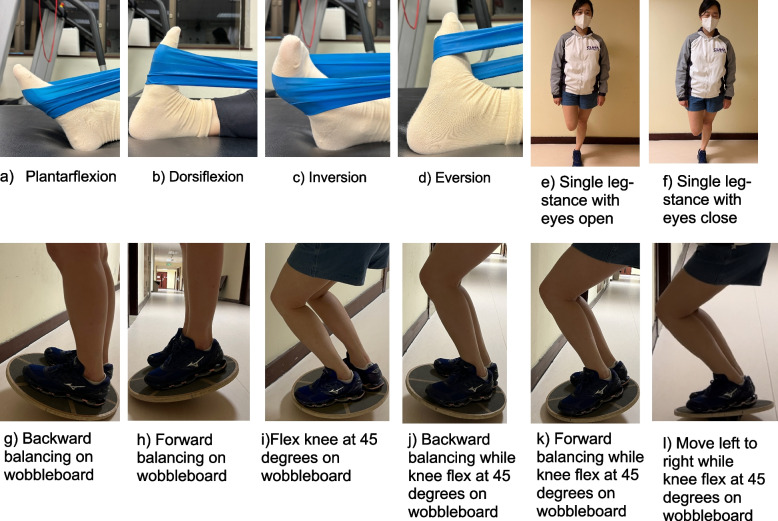
Resistance training and balance training

Between the fourth and eighth week, participants will advance to flexing their knees on the wobble board and repeating the same exercise performed during the first to third week. Each repetition will last for 30 s, with a total of 5 repetitions. A rest period of 20 s will be observed between each repetition. Participants must be in a unilateral stance with eyes opened and closed for 7 s and 4 s, respectively, in sets of 5 with a 10-s rest. Concurrent with the balance training, participants will participate in the ankle muscle strengthening exercise using resistant bands (Thera-bands). Participants are required to sit on the floor with one end of the tubing tied around the treatment table and the other wrapped around the affected foot’s involved metatarsals. Participants will perform different ankle rotations using resistant bands, such as eversion, inversion, plantarflexion, and dorsiflexion. Regardless of the tension of the band, participants are required to stretch the band at 170% of the resting length. Exercise progression will include increasing the number of sets from 1 to 3 for 8 to 10 repetitions each—lastly, stretching of the ankle joint during the cooling session. The major exercise regime can be seen in Fig. 3.

### Criteria for discontinuing or modifying allocated interventions {11b}

A participant can opt out of the study at any time point. Treatment will be halted immediately, and required treatment will be provided whenever there is an adverse reaction, including pain, swelling, and discomfort. Consultations with the orthopedic surgeons will be made available via the research clinic from the Department of Orthopaedics and Traumatology, CUHK.

### Strategies to improve adherence to interventions {11c}

The opening hours of the Sports Performance and Biomechanics Laboratory will be extended to evening hours for the convenience of participants and to facilitate their participation. In addition, participants will be asked if there is any difficulty with the exercise regime whenever they come for the PEMF therapy.

### Relevant concomitant care permitted or prohibited during the trial {11d}

Participants are allowed to participate in their usual activities; no restrictions or changes to their daily activities will be required.

### Provisions for post-trial care {30}

Participants will have access to the research clinic from the Department of Orthopaedics and Traumatology, CUHK, if they experience any adverse reaction.

### Outcomes {12}

#### Primary outcome

**Fig. 4:**
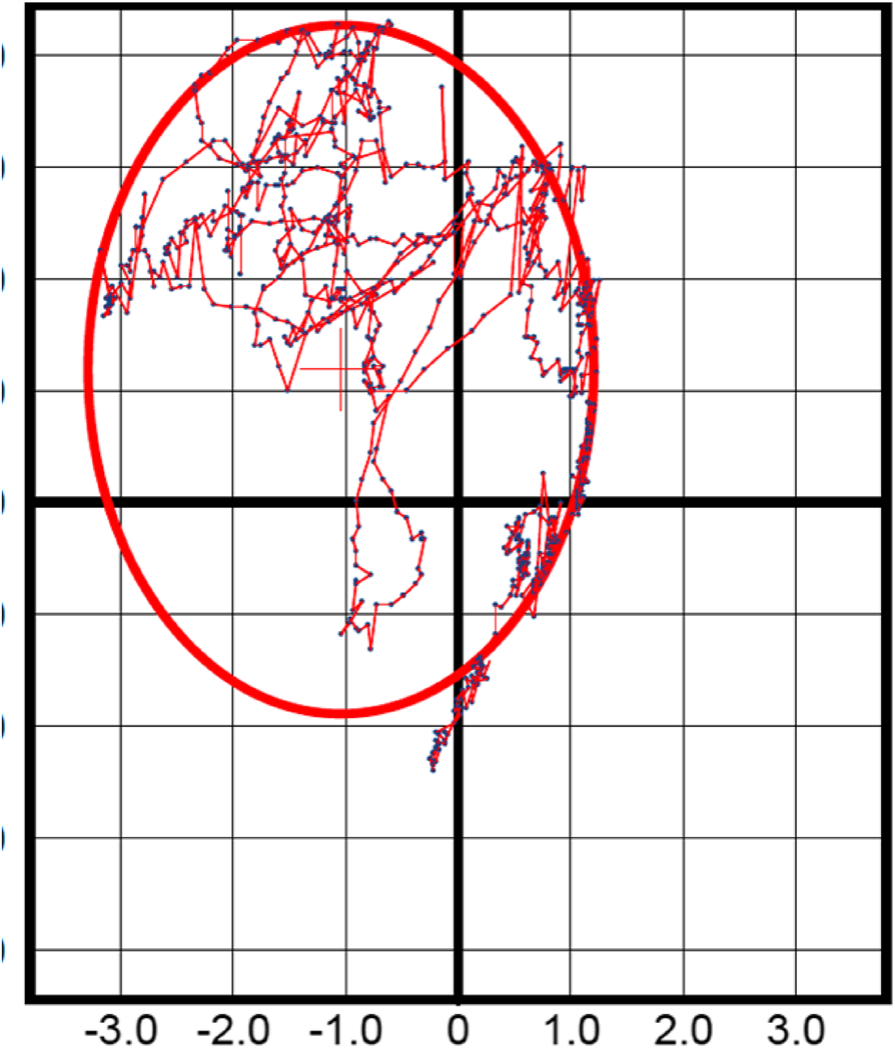
A TekScan result for anterior–posterior and mediolateral direction

Single leg standing test will be assessed on the TekScan MatScan® pressure mat model 3150 (TekScan Inc, South Boston, USA) to evaluate the postural control stability. The TekScan MatScan® is a low-profile floor mat (5 mm thick) and consists of 2288 resistive sensors (1.4 sensors/cm^2^) with a sampling frequency of 40 Hz and has been well-validated in other populations [32]. The mat will provide measures of anterior–posterior and mediolateral sway parameters described as the area and direction of the sway, distance and the direction traveled by the center of pressure (COP), and the variability of the distance traveled by COP. The Sway Analysis Module (SAM™) software will be used to analyze the sway data as seen in Fig. 4.

On the pressure mat, participants will perform one practice trial and two trials of single limb stance on each leg with eyes opened and closed for 20 s, respectively. Participants will be required to stand on one leg unassisted (barefooted) and hands on the hips. This will be repeated for the non-affected leg as well. Before the commencement of the study, the assessor will undergo training, and the same assessor will oversee the experiment.

#### Secondary outcomes

##### Functional score

Cumberland Ankle Instability Tool (CAIT) score is a valid and reliable 9-item instrument used to identify self-perceived ankle instability. The instrument is scored on a 0 (worst) to 30 (best) scale, where a cut-off point of ≤ 24 denotes the presence of instability [33].

##### Dynamic balance

The Y balance test (YBT) evaluates the dynamic balance control in the CAI population. This test requires the integration of sensory inputs from visual, vestibular, and somatosensory pathways resulting in controlled motor outputs that recruit specific motor inputs. Participants must maintain unilateral stance, with their hands on the hips, balance on the involved limb, and reach with the uninvolved limb in the anterior, posteromedial, and posterolateral directions as far as possible [34]. The trial will not be counted and repeated if the participants lift their heels, fail to maintain their balance, remove their hands from their hips, or return to the starting point. Participants will complete 4 times of the trial and 3 analysis trials in each direction of the limb. The maximum distance in each direction, mainly, anterior, posteromedial, and posterolateral will be taken and divided by the limb length (LL) and multiplied by 100 [35]. LL will be measured using a flexible measuring tape from the anterior superior iliac spine to the medial ankle. The YBT composite (%) is calculated by the sum of three reach directions divided by 3 times the LL multiplied by 100.

##### Eversion muscle strength

Peroneal longus (PL) contribute greatly to ankle eversion; ankle eversion muscle strength will be measured using an isometric digital handheld dynamometer (MicroFET2, Manual Muscle Testing) under a standardized protocol [36, 37]. Participants will be placed in a prone position, and the ankle posture will be anatomically neutral. A handheld dynamometer will be placed on the lateral metatarsal head for the eversion testing. The patient will exert muscle strength, and the examiner will counteract with resistance for 5 s and measure the muscle strength isometrically. The healthy ankle will be examined first, followed by the injured ankle. The test will be performed thrice, with the highest value recorded in kilograms. The maximum strength (kg) will be taken as the participant’s peak strength and the result will be normalized by the body weight (kg). Both the injured and contralateral limbs will be tested.

##### Dorsiflexion range of motion

Weight-bearing lung test is based on the knee-to-wall principle and is a valid and reliable assessment that measures the DROM in CAI population. The procedure requires the participant to first perform a lunge in for the untested limb where the knee flexes to a point for the anterior knee to touch the wall with the tested heel remaining firmly on the floor. The maximum distance is determined from the tip of the great toe to the wall, nearest to 0.1 cm, using a long ruler [38]. Upon successful contact of the knee to the wall, the participant will progress backward in 1 cm increments from the wall until either the heel or the knee can no longer be maintained during the lunge. The participant will be given 3 attempts to achieve the greatest distance between the large toe and the wall. Both the injured and contralateral limb will be tested.

##### Lateral step-down test (LSDT)

LSDT is a clinical measurement that evaluates dynamic postural stability deficits and potential lower extremity injury. It measures the coordination of the lower limb joints, neuromuscular control, strength, pelvic stability, and range of motion [39]. A 30-cm high step will be used for the test, and the participant will be instructed to align the stance limb foot with the lateral edge of the step with their hands on their hips. Participants will also be instructed to descend the step with the tested heel gently touching the TekScan MatScan® pressure mat model 3150 (TekScan Inc, South Boston, USA), with the contralateral foot lifted off the platform. The trial will not be considered successful when the hands were not placed at the iliac crest during the starting point, the testing leg touches the pressure mat before the opposite leg left the step, or the participant jumped instead of stepping down. Participants are required to balance on the contralateral heel for 20 s, and the COP will be captured by the Sway Analysis Module (SAM™) software. Both the injured and contralateral limb will be tested; participants will repeat the test 3 times on each leg.

##### Ultrasonographic outcomes

A standardized ultrasound examination is used to evaluate the peroneal longus (PL) in a reliable and repeatable manner. It will be performed during baseline assessment and all follow-up visits. The overall ultrasound examination has shown high intra-reliability (ICC = 0.925, *p* < 0.01) and inter-rater reliability (0.947, *p* < 0.01) and was conducted together with a researcher (HX) who has 5 years of experience with ultrasound imaging.

##### Cross-sectional and thickness of the peroneal muscle

Musculoskeletal ultrasound scanning will be performed in the supine position with the probe at 25% of the distance between the fibula head and lateral malleolus. Peroneus longus (PL) and peroneus brevis (PB) were scanned together transversely by positioning the long axis of the probe perpendicular to muscle fibers to obtain the image of the cross-sectional area (CSA) [40]. CSA will be taken as the area of muscle perpendicular to its longitudinal direction along the line between the fibular head and lateral malleoli. The probe will be rotated into a longitudinal orientation for the thickness of the peroneal longus (PL). The thickness will be defined as the distance between each peroneal muscle’s aponeuroses (white fibrous tissue) [40].

##### The elasticity of the peroneal muscle

In addition, the shear elastic modulus of a muscle will be measured using ultrasonic shear wave elastography (Aixplorer, Supersonic Imagine, France) with a linear array probe. The region of interest (ROI) will be set near the center of each muscle. The analysis area will be a 5-mm diameter circle at the center of ROI. The shear elastic modulus will be measured three times per muscle, and the average of the three measured values will be used for further analysis. The shear elastic moduli of the PL and PB will be measured at proximal 25% from the head of the fibula to the lateral malleolus. For each Q-box, the maximum, minimum, and mean (in kPa) of the stiffness of the muscle are measured [41].

Other patient-reported outcome measures (PROM) will be employed to assess overall health and foot and ankle-related symptoms, pain, physical activity level, and health-related quality of life. Foot and Ankle Ability Measure (FAAM) will be used to assess the foot and ankle functional limitations among CAI individuals. The FAAM consists of 29 items, scored between 0 and 4, divided into two subscales: activities of daily living (21 items) and sports (8 items) [42]. For score analysis, the percentage of each subscale is used separately. According to the International Ankle Consortium, values of the day living activities < 90% and < 80% of the sports subscales of the FAAM score were considered unsatisfactory. Manchester-Oxford foot and ankle questionnaire (MOXFQ) is a 16-question self-reported score designed to evaluate the impact of foot and ankle problems on a person’s quality of life. The dimensions comprise 3 subscales: walking/standing problem (7 items), foot pain (5 items), and issues related to social interaction (4 items). After that, all three domains of the MOXFQ can be summed up and converted to a metric from 0 to 100 to create a summary index score (MOFXQ index) [43]. The pain score will be evaluated using the visual analog scale (VAS) ranging from 0 to 100. For the pain score, the higher the score indicates, the more severe the pain. The Short Form-36 questionnaires (SF-36) will be used to evaluate the health-related quality of life [44]. The International Physical Activity Questionnaire will be used to evaluate the physical activity levels [45]. All primary and secondary outcomes will be evaluated at baseline, 4th week, 8th week, 3rd month, 6th month, and 12th month follow-up.

### Participant timeline {13}

The schedule of study outcomes is assigned in Table 1.

**Table 1:**
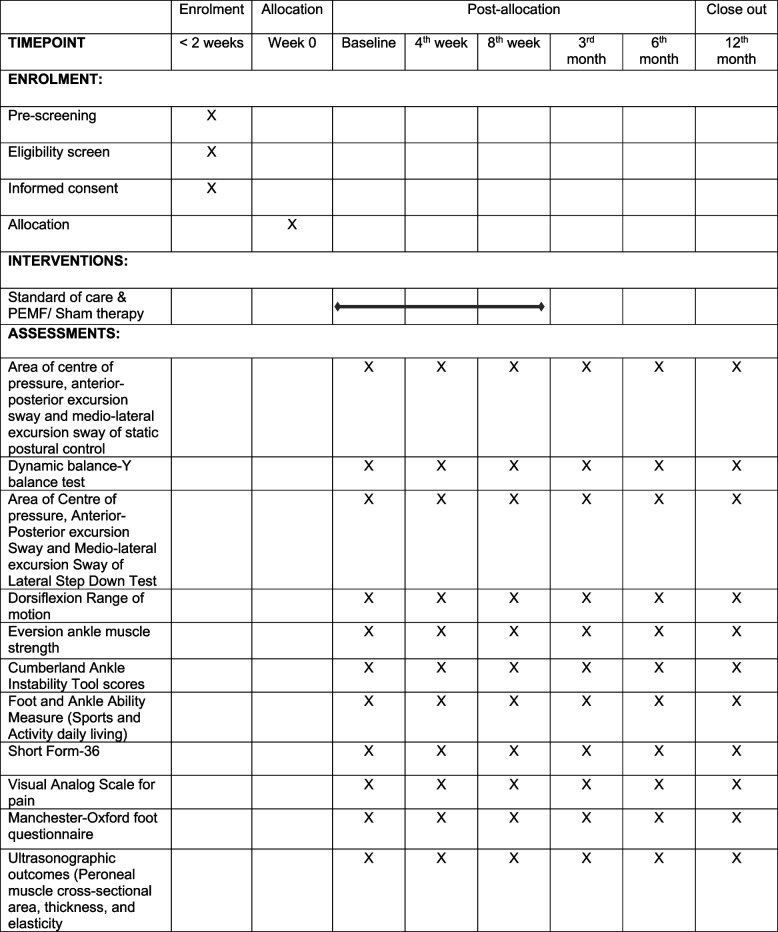
The enrolment, intervention, assessment, and visit schedule for the participants

The X indicates at which point of the trial the respective assessments will take place

### Sample size {14}

Using the G*Power software (Germany) [46], a sample size of 48 (*n* = 24 per group, 1:1 allocation) is calculated based on the highest attrition rate of 40% at 3rd month follow-up of the study to retain a sample size of *n* = 28, according to similar research [47]. The calculation is according to the *F* test-repeated measures ANOVA, between factors, with an effect size of 0.3, an alpha level of 0.05, (1 − *β* err prob) = 0.8 to detect a significant change of the area of COP from 42.0 (± 23.6) to 25.4 (± 8.2) [48].

### Recruitment {15}

Participants will be principally recruited from the Department of Orthopaedics and Traumatology, Prince of Wales Specialist Outpatient Clinic. The orthopedic surgeons will screen the participants based on their clinical symptoms and explain the details of the study to the participants. Furthermore, the recruitment information for the trial will be delivered to various sports teams and through the internal mass-mail system at CUHK. They will be referred to the research team for eligibility assessment at the Sports Injury and Biomechanics Laboratory should they want to join the trial.

### Assignment of interventions: allocation

#### Sequence generation {16a}

Participants will be randomly assigned to a unique radiofrequency identification (RFID) card number in a 1:1 ratio by an online research randomizer (https://www.randomizer.org/). The unique RFID card is provided by the Quantum TX. Each allocation will be tagged with a unique RFID card generated by the chief engineer from Quantum TX and is recognized by the PEMF machine.

#### Concealment mechanism {16b}

The randomization will be conducted using an online research randomizer by the chief engineer at Quantum TX and the random order of the treatment allocation will only be shared with the biostatistician, a third party based at the CUHK who is not involved in the recruitment process. The randomization will not be disclosed to either the participants or the trial assessors throughout the intervention period. The participants will be randomly assigned to a unique and identical RFID card in which the PEMF or the sham group are randomly allocated to the RFID card. This is to ensure that the participants and the assessors are both blinded to the unique RFID cards containing the treatment types.

#### Implementation {16c}

The chief engineer of Quantum TX will generate the randomization, and the randomization results will only be shared with the biostatistician at the CUHK. The research team and the orthopedic surgeons will recruit the participants. The Ph.D. student will assist in the assessment and assign the participants to the interventions. At the end of the intervention, the biostatistician will reveal the randomization results to the research team and orthopedic surgeons for statistical analysis.

### Assignment of interventions: blinding

#### Who will be blinded {17a}

The assessors and orthopedic surgeons will be blinded to the assignment of the interventions. Since the PEMF therapy will not elicit heat or sensation, the participants will also be blinded to the treatment allocation. The randomization results will only be disclosed to the assessors and the orthopedic surgeons after the PEMF intervention has ended.

#### Procedure for unblinding if needed {17b}

The procedure will only be unblinded if there is an occurrence of an adverse event. The adverse event will be reported to the Joint Clinical Research Ethical Committee of the CUHK and the New Territories East Cluster of the Hospital Authority.

### Data collection and management

#### Plans for assessment and collection of outcomes {18a}

Demographic data and injury history will be collected during screening and before the recruitment. Participants who have fulfilled the eligibility criteria will be randomly allocated into the treatment groups. This is followed by the collection of the outcomes as listed in “Results” at baseline and different time points.

#### Plans to promote participant retention and complete follow-up {18b}

The office hours of the Sports Performance and Biomechanics Laboratory, Prince of Wales Hospital, will be extended to the evening to accommodate the participants’ working hours for the PEMF therapy and assessments.

#### Data management {19}

The collected data will be entered into Excel and SPSS files. They will all be password-protected and destroyed 5 years after publication in Ph.D. thesis and peer-reviewed journals. The Ph.D. student will be responsible for the data collection, data entry, and data analysis under the guidance of the biostatistician. Double data entry and range checks for data values will promote data quality.

#### Confidentiality {27}

All online data relating to the participants will be password-protected on the laboratory’s computer, and hard copies (i.e., consent forms and data sheets) will be placed in files and kept in a locked cabinet at the Sports Performance and Biomechanics Laboratory. Only the research team in charge of the study will have access to the information. Participants will not have any access to the data set. There is no plan to share the data collected with other organizations.

#### Plans for collection, laboratory evaluation, and storage of biological specimens for genetic or molecular analysis in this trial/future use {33}

There are no plans to collect any biological specimens for genetic or molecular analysis in this trial.

### Statistical methods

#### Statistical methods for primary and secondary outcomes {20a}

SPSS version 26.0 will be used to analyze the primary and secondary outcomes based on the intention to treat (ITT) principle. ITT analysis will be performed using all randomized patients, including data for those terminated or lost to follow-up. During the process, assumptions will be checked, and remedial measures will be employed. The normality of the data will first be determined using the Kolmogorov–Smirnov test or the Shapiro–Wilk test (*p* > 0.05) to test for the normality of the data distribution. Other graphical methods such as Q-Q plots or histograms will also be used for visual inspection of the distribution and to assess the normality. If skewness and heteroscedasticity are present, transformation methodology such as Logarithm may be applied to make the distribution more symmetric and stabilize the variance. Baseline data and clinical variables between both groups will be compared using independent Student’s* t*-tests (parametric)/Mann–Whitney *U* test (non-parametric) for continuous data and the χ^2^ of independence for categorical data, respectively. Descriptive statistics will be presented in either mean (SD) or frequency (%). Thereafter, a repeated measure analysis of variance (RMANOVA) will be used to measure the time/exposure effects of muscle strength, Y balance test, LSDT, SLT, DROM, and results of the questionnaire between baseline, week 4, week 8, 3rd month, 6th month, and 1-year follow-up. Analysis of covariance (ANCOVA) will be conducted for baseline characteristics and prognostic factors as covariates and compared for group differences in different time points. Repeated-measure ANOVA and ANCOVA will be applied for all participants who have completed the assessments post-treatment. Bonferroni correction will be performed for multiple comparisons. Sensitivity analysis will be conducted by comparing the results of the imputation method to complete-case and available data analyses. The significant level will be set at 0.05.

#### Interim analyses {21b}

Interim results will be performed when 20% of the participants have completed the 8-week PEMF treatment. The interim analysis ensures that the study is feasible and safe during the study period.

#### Methods for additional analyses (e.g., subgroup analyses) {20b}

No subgroup analysis will be performed.

#### Methods in analysis to handle protocol non-adherence and any statistical methods to handle missing data {20c}

Intention to treat analysis will be deployed to include all study participants regardless of their treatment allocation and whether the intervention for them has been completed. Regardless of the adherence to the intervention or withdrawal, they will be recorded by the Ph.D. student weekly. Missing data will be checked, and multiple imputations and sensitivity analysis will be employed assuming that the missing data are random.

#### Plans to give access to the full protocol, participant-level data, and statistical code {31c}

The protocol has been registered on the ClinicalTrials.gov (ID: NCT05500885). The study data will be available from the principal investigator upon reasonable request.

## Oversight and monitoring

### Composition of the coordinating center and trial steering committee {5d}

The principal investigator will monitor the progress of the study. The trial steering committee will include the research members and Ph.D. students who will participate in the daily support of the trial, ranging from participant recruitment, data collection, and management.

### Composition of the data monitoring committee, its role and reporting structure {21a}

The trial steering committee will include orthopedic surgeons, a Ph.D. student, and a biostatistician. The Ph.D. student will participate in the data collection and analysis under the guidance of orthopedic surgeons and biostatisticians. The biostatistician will monitor the data collection and perform interim results. The ethical committee requires no independent data committee. Furthermore, the PEMF treatment imposes little side effects as a conventional treatment for various musculoskeletal disorders. Therefore, it is a low-risk study.

### Adverse event reporting and harms {22}

Based on the Food and Drug Administration approval, PEMF therapy has been declared safe for users [49]. Nonetheless, the research team will regularly monitor adverse events in every visit. Upon any adverse events, they will be reported to the trial steering committee immediately. The participant will be withdrawn from the study immediately and referred to the outpatient clinic at the Prince of Wales Hospital.

### Frequency and plans for auditing trial conduct {23}

An independent auditor from the ethical committee will conduct the trial annually throughout the research project period. The trial steering group meets weekly to update the principal investigator on the research progress. The principal investigator will complete a standardized progress report to the ethical committee annually.

### Plans for communicating important protocol amendments to relevant parties (e.g., trial participants, ethical committees) {25}

There are currently no plans to modify the protocol. Any amendments to the protocol will be submitted by the principal investigator to the ethical committee before the implementation. The trial participants will also be informed if there are any deviations from the protocol.

### Dissemination plans {31a}

The interim findings of the research project will be disseminated through local conferences such as the Hong Kong Orthopaedic Association Annual Congress. The finalized results will be published in peer-reviewed journals and published at international conferences such as the Asia–Pacific Knee Arthroscopy and Sports Medicine (APKASS) Congress.

## Discussion

CAI is a complex and heterogeneous condition that debilitates quality of life. The phenomenon is characterized by the deficit in sensory and motor functions within the sensorimotor system caused by the deafferentation of the damaged sensory receptors. Consequently, the sensory information from the sensorimotor system has been reweighted from various somatosensory sources to achieve postural control stability.

The exact reason that a significant portion of patients develop CAI after LAS is not well understood. Arthrogenic muscle inhibition (AMI) has been suggested as one of the major reasons why individuals do not recover fully from an LAS [50]. Individuals with CAI exhibited altered neuromuscular firing and motor recruitment patterns, which cause a delay in peroneal activation during an inversion perturbation [51, 52]. Hence, this may lead to persistent muscle weakness and reduced postural control stability [50, 53, 54]. Especially the impairment of postural control stability which has been frequently reported after an index LAS increasing the risk of noncontact LAS during dynamic activities causing symptoms of CAI [55–57].

Although the current SC with the best efficacy level is muscle strengthening and proprioception training, it is limited by the heavy loading or resistance which may induce pain or recurrent injuries [58]. Furthermore, SC treatment may not fully activate muscle contraction which causes persistent muscle weakness and delayed peroneal muscle activity represented by the increased area of COP during single leg stance test, an evaluation of postural control stability [59]. Therefore, investigating novel treatments to improve peroneal muscle weakness and postural control stability remains a priority to function in the CAI population [60].

Previous collaborative work coupled with other supporting evidence have shown that short exposure to low-energy PEMF therapy improved muscle regeneration and improved systemic metabolism/inflammation via paracrine means in cells [21, 61–63], animal studies [64, 65], and human studies [17, 19]. Meanwhile, evidence have shown that PEMF therapy can significantly alleviate pain and swelling in acute LAS individuals [66, 67]. However, there is limited data on the application of PEMF therapy in the areas of the CAI spectrum. Recent evidence has shown that PEMF therapy may improve muscle contraction by increasing muscle activation and reducing electromechanical delay independently from the neural pathway, which resulted in a shortened recovery time [20] . Therefore, we proposed that the application of PEMF therapy in addition to SC may improve both postural control sway and peroneal muscle weakness in CAI individuals through a similar pathway and less AMI, which helps in muscle gains. With the PEMF regime being well-established within our team, it can be exploited as a novel adjunct treatment on top of the SC for the CAI population.

This current trial protocol may demonstrate the effectiveness of PEMF therapy in improving PCS, dynamic balance, eversion strength, functions, and pain since the rehabilitation effect of PEMF therapy and SC on CAI patients has not been explored yet. Therefore, making this protocol a significant contribution to the assessment of the PEMF therapy as a treatment among CAI individuals. In the short term, this trial would benefit individuals with CAI who experienced persistent peroneal muscle weakness and impaired PCS. In both the medium and long term, this would potentially lead to a better return to play and reduce the risk of re-injury, which can be a burden to the health system and society due to additional treatments and time off from work. Regardless of the findings, the information is useful to both CAI individuals who suffer from peroneal muscle weakness and poor PCS, and an indication to the clinicians that PEMF therapy may be a potential treatment to render improvement.

## Trial status

The enrolment of the study started on 1st June 2022. Recruitment is expected to be completed by the end of December 2024.

## Data Availability

The availability of data and materials is available upon reasonable request.

## Abbreviations

ANOVA: Analysis of variance
AMI: Arthrogenic muscle inhibition
CAI: Chronic ankle instability
CAIT: Cumberland Ankle Instability Tool
COP: Center of pressure
CUHK: Chinese University of Hong Kong
CSA: Cross-sectional area
DROM: Dorsiflexion range of motion
FAAM: Foot and Ankle Ability Measure
IPAQ: International Physical Activity Questionnaire
ITT: Intention to treat
LAS: Lateral ankle sprain
LSDT: Lateral step-down test
PB: Peroneus brevis
PEMF: Pulse electromagnetic field
PGC-1α: Peroxisome proliferator-activated receptor-gamma coactivator
PROM: Patient-reported outcome measures
PL: Peroneus longus
RFID: Radiofrequency identification
ROI: Region of interest
SF-36: Short Form-36
SC: Standard care
